# Interleukin-18 Is a Potential Biomarker to Discriminate Active Adult-Onset Still’s Disease From COVID-19

**DOI:** 10.3389/fimmu.2021.719544

**Published:** 2021-07-23

**Authors:** Po-Ku Chen, Joung-Liang Lan, Po-Hao Huang, Jye-Lin Hsu, Ching-Kun Chang, Ni Tien, Hui-Ju Lin, Der-Yuan Chen

**Affiliations:** ^1^ Rheumatology and Immunology Center, China Medical University Hospital, Taichung, Taiwan; ^2^ College of Medicine, China Medical University, Taichung, Taiwan; ^3^ Translational Medicine Laboratory, China Medical University Hospital, Taichung, Taiwan; ^4^ Rheumatic Diseases Research Center, China Medical University Hospital, Taichung, Taiwan; ^5^ Graduate Institute of Biomedical Sciences, China Medical University, Taichung, Taiwan; ^6^ Department of Laboratory Medicine, China Medical University Hospital, Taichung, Taiwan; ^7^ Ph.D. Program in Translational Medicine and Rong Hsing Research Center for Translational Medicine, National Chung Hsing University, Taichung, Taiwan

**Keywords:** galectins, cytokine profile, ferritin, COVID-19, adult-onset Still’s disease (AOSD)

## Abstract

**Background:**

Hyperinflammation with dysregulated production of galectins and cytokines may develop in COVID-19 or adult-onset Still’s disease (AOSD). Given the similar clinical features in both diseases, it is necessary to identify biomarkers that can differentiate COVID-19 from AOSD. However, the related data remain scarce currently.

**Methods:**

In this cross-sectional study, plasma levels of galectin-3, galectin-9, and soluble TIM-3 (sTIM-3) were determined by ELISA in 55 COVID-19 patients (31 non-severe and 24 severe), 23 active AOSD patients, and 31 healthy controls (HC). The seropositivity for SARS-CoV-2 was examined using an immunochromatographic assay, and cytokine profiles were determined with the MULTIPLEX platform.

**Results:**

Significantly higher levels of galectin-3, galectin-9, IL-1β, IL-1Ra, IL-10, IFN-α2, IL-6, IL-18, and TNF-α were observed in severe COVID-19 and active AOSD patients compared with HC (all p<0.001). AOSD, but not COVID-19, showed significantly higher IFN-γ and IL-17A compared with HC (both p<0.01). Moreover, active AOSD patients had 68-fold higher IL-18 levels and 5-fold higher ferritin levels than severe COVID-19 patients (both p<0.001). IL-18 levels at the cut-off value 190.5pg/mL had the highest discriminative power for active AOSD and severe COVID-19, with AUC 0.948, sensitivity 91.3%, specificity 95.8%, and accuracy of 91.5% (p<0.005). Multivariate regression analysis revealed IL-18 as a significant predictor of active AOSD (p<0.05).

**Conclusion:**

Active AOSD patients share features of hyperinflammation and cytokine storm with severe COVID-19 patients but possess a distinct cytokine profile, including elevated IL-18, IL-6, IFN-γ, and IL-17A. IL-18 is a potential discriminator between AOSD and COVID-19 and may significantly predict active AOSD.

## Introduction

Globally, more than 120 million people had been infected with severe acute respiratory syndrome coronavirus 2 (SARS-CoV-2), and more than 2 million people had died of coronavirus disease 2019 (COVID-19) by March 2021. COVID-19 commonly manifests as fever, myalgia or fatigue, respiratory symptoms, and may cause rapid deterioration of pulmonary involvement (1, 2). In laboratory data, COVID-19 patients tend to have elevated acute phase reactants and ferritin levels, and lymphocytopenia (1, 2). In response to SARS-COV-2 infection, cytokine production may be rapidly dysregulated, leading to a systemic hyperinflammation status, the so-called cytokine storm (3, 4). A variety of inflammatory or anti-inflammatory cytokines, such as interleukin (IL)-1β, IL-6, IL-8, IL-10, and interferon (IFN)-γ, were elevated in severe COVID-19 patients (3, 4). Coperchini et al. also mentioned that the IL-6/CXCL10/macrophages axis is crucial in driving the cytokine storm (5). Meanwhile, the expression levels of NOD, LRR, and pyrin domain-containing protein 3 (NLRP3)-inflammasome signaling molecules were also increased, which parallel the severity of COVID-19 (6) and may further induce multisystem inflammatory syndrome. Besides, impaired Type-I IFN responses to SARS-CoV-2 in the initial stage may lead to a cytokine storm (7). Several previous studies, including a systemic review and meta-analysis, revealed that the occurrence of cytokine storm is associated with COVID-19 severity and mortality (3, 4, 7–10). Therefore, early identification and optimal treatment of cytokine storms are pivotal in improving disease outcomes (9, 10).

Adult-onset Still’s disease (AOSD), an autoinflammatory disorder, is characterized by fever, rash, arthralgia or arthritis, myalgia, liver dysfunction, multi-systemic involvement, increased acute phase reactants, hyperferritinemia, and even life-threatening complications such as macrophage activation syndrome (MAS) (11–14). It is also marked by elevated proinflammatory cytokines, including IL-1β, IL-6, IL-8, IL-17, IL-18, and IFN-γ (15–18), which are involved in a cytokine storm. We have similarly revealed an elevated expression of NLRP3-inflammasome with overproduction of IL-1β and IL-18 in AOSD patients (19). Accordingly, inhibitors to IL-1, IL-6, and IL-18 have been shown effective in AOSD treatment (20–22).

There are several similarities in clinical manifestations between COVID-19 and AOSD. Both diseases often manifest as fever, myalgia or fatigue, elevated acute phase reactants and ferritin levels, liver dysfunction, and lymphocytopenia. During the COVID-19 pandemics, clinicians are eager to find biomarkers that can differentiate between AOSD and COVID-19, particularly in febrile patients with elevated C-reactive protein (CRP) or hyperferritinemia (23). Accurate discrimination is crucial for the early protection, prevention of spreading, and precision treatment.

Galectins play an important role in regulating immune reactions and inflammatory responses (24). Galectin-3 (Gal-3), a 30-kDa glycan-binding protein expressed on various immune cells, is involved in both innate and adaptive immunity (24, 25). Gal-3 can act as a modulator of cytokine expression in immune cells and an orchestrator of the damage associated with the molecular pattern (DAMP) (26). As shown in single-cell analysis, Gal-3 levels in the myeloid cells from severe COVID-19 patients were significantly higher than those from mild disease (27). Patients with severe COVID-19 had significantly higher Gal-3, TNF-α, IL-1β, and IL-6 than those with moderate disease (27–29). Besides, Gal-3 levels in AOSD patients were elevated and correlated with NLRP3-inflammasome downstream cytokines IL-1β and IL-18 (30). Galectin-9 (Gal-9), a ligand of T cell immunoglobulin and mucin-containing-molecule-3 (TIM-3), is expressed on type 1 helper T (Th1) and Th17 cells and provides inhibitory signals (31). It regulates pro-inflammatory T cell responses through the Gal-9/TIM-3 pathway and induces apoptosis of Th1 or Th17 cells (31, 32). Gal-9 levels were higher in COVID-19 patients than in healthy subjects (28). Fujita et al. also revealed that Gal-9 levels were elevated and correlated with AOSD activity (33).

Although an initial cytokine storm or hypercytokinemia could occur in both COVID-19 and AOSD, the phenotype or immune heterogeneity of the cytokine storm in AOSD may differ from COVID-19 (34). Recently, Meng et al. used the databases to compare cytokine profiles between AOSD and COVID-19, and revealed higher IL-6 and IL-10 in severe COVID-19 than in AOSD (35). Due to the potential variations among database studies, a direct comparison of the circulating galectins levels and cytokine profiles between COVID-19 and AOSD would more clearly illustrate their differences.

This pilot study investigated the differences in the circulating Gal-3, Gal-9, sTIM-3, ferritin levels, and cytokine profiles between COVID-19 and active AOSD patients. We also identified the potential biomarkers to discriminate active AOSD from severe COVID-19.

## Methods

### Patients and Study Design

Given a low prevalence of COVID-19 infection in Taiwan (slightly more than 1000 cases, 4.0/100,000 assessed on March 31, 2021) (36), twenty-five plasma samples obtained from the donors with laboratory-confirmed COVID-19 were purchased from BocaBiolistics (Pompano Beach, FL, USA) (SOP 10-00414 Rev E [De-linking specimens]). National Health Research Institute sponsored other 30 blood samples obtained from Chinese patients with laboratory-confirmed COVID-19. Confirmed COVID-19 was defined as a positive result of polymerase-chain-reaction assay of nasal or pharyngeal swab specimens. According to the report of the WHO-China Joint Mission on COVID-19, the severity of COVID-19 patients was divided into mild (constitutional symptoms without pneumonia), moderate (COVID-19 pneumonia), and severe (severe dyspnea, adult respiratory distress syndrome requiring mechanical ventilation, shock, other organs failure that requires intensive care, or mortality) (37). Mild and moderate COVID-19 are considered non-severe.

In this cross-sectional study, twenty-three active AOSD patients fulfilling the Yamaguchi criteria (38) and having a negative result of IgG/IgM for SARS-CoV-2 were enrolled. Systemic disease activity was assessed with a modified Pouchot score (39), with active AOSD defined as systemic activity scores higher than four (40). Thirty-one healthy volunteers who had no rheumatic disease or anti-SARS-CoV-2 IgG/IgM positivity were enrolled as healthy control subjects.

### Determination of SARS-CoV-2 Antibody-IgG/IgM

Seropositivity for SARS-CoV-2 was determined using an immunochromatographic assay (Guangzhou Wondfo Biotech Co., Ltd., Guangzhou, P. R. China). Ten μl of plasma samples were added to the wells, and then 80μl buffer solution was added to the buffer wells. The results were interpreted after 15 minutes of incubation.

### Determination of Plasma Levels of Gal-3, Gal-9, and Soluble TIM3 (sTIM-3)

Ten ml of whole blood was collected in tubes containing EDTA (BD Biosciences, San Jose, CA, USA), and were centrifuged at 2,000 rpm for 10 min. Plasma samples were stored in aliquots at −80°C until use. Gal-3 (Cat#DY1154), Gal-9 (Cat#DY2054), and sTIM-3 (Cat#DY2365) were measured using Duoset-ELISA Kit (R&D Systems., Minneapolis, MN, USA) according to the manufacturer’s instructions. Briefly, the 96-well microplate was coated with 100μl diluted capture antibody in each well overnight at room temperature (RT), then was incubated with 1% BSA in PBS (Reagent Diluent, 200μl) for 1 hr at RT. A 100μl of sample (5X diluted in Reagent Diluent) were added to each well and incubated for 2 hrs. at RT. Each well was incubated with the 100μl of diluted detection antibody for 2 hrs. at RT, and then 100μl of the Streptavidin-HRP (200X dilution) was added to each well at RT with incubation of 20 minutes and avoided in direct light. Subsequently, each well was washed with PBS containing 0.1% Tween20 using a manifold dispenser, and then 100μl of Substrate Solution were added to each well with an incubation time of 20 minutes at RT. Finally, 50μl of Stop Solution were added and Absorbance was measured at 450nm or 540nm by the BioTek Synergy HT plate reader (BioTek Instruments, Winooski, VT).

### Determination of Plasma Levels of Ferritin and Cytokine Profile

Plasma levels of light-chain ferritin were measured with ELISA (Cat# MBS167446 Mybiosource, San Diego, CA, USA) according to the manufacturer’s instructions. Briefly, 50μl standard solutions and 40μl plasma samples with 10μl anti- light-chain ferritin antibody were added to strip-wells. Then, 50μL streptavidin-HRP was added to each well. The plate was covered with a sealer and incubated for 60min at 37°C, and then washed with 200μl washing buffer for 5 times using a manifold dispenser. Mixture of 50μl substrate solution A and 50μl substrate solution B was added to each well and then incubated for 10 minutes at 37°C in the dark. Finally, 50μl Stop Solution was added to each well. Absorbance was measured at 450nm by the BioTek Synergy HT plate reader. Given the potential variability in cytokines quantification across the platform, plasma levels of IFN-α2, IFN-γ, IL-1β, IL-1 receptor antagonist (IL-1Ra), IL-6, IL-10, IL-17A, IL-18, and TNF-α were determined by magnetic multiplex using a MULLIPLEX^®^ Human Cytokine/Chemokine/Growth Factor Panel A (Cat# HCYTOMAG-60K-16) according to the manufacturer’s instructions (Milliplex MAP kits, EMD Millipore, Billerica, MA, USA).

### Statistical Analysis

We performed a chi-squared test to examine the difference of distribution in sex among the four groups. The Kruskal-Wallis test with a *post-hoc* Dunn’s test was used to compare Gal-3, Gal-9, sTIM-3, ferritin, and cytokine profiles among multiple groups. The Benjamini–Hochberg procedure with a false discovery rate 0.05 was used to adjust for multiple testing. The missing values were excluded from the statistical analysis. A multivariate logistic regression model was used to evaluate cytokine profiles for discriminating AOSD from COVID-19. The receiver-operating characteristic (ROC) curve analysis was performed to determine the area under the ROC curve (AUC), sensitivity, and specificity using MedCalc v.14. A *p*-value<0.05 was considered significant. A two-sided probability of less than 0.05 was considered significant.

## Results

### Clinical Characteristics of COVID-19 Patients and AOSD Patients

As illustrated in **Table 1**, patients with COVID-19 (mean ± SD, 46.8 ± 16.0 years), particularly severe COVID-19 (52.1 ± 15.7 years), were older than those with active AOSD (42.6 ± 13.5 years), with male predominance in COVID-19 patients compared with AOSD (52.7% *vs*. 17.4%, p<0.05). Fever was the most common manifestation in both active AOSD and COVID-19 (91.3% and 81.8%, respectively). Myalgia and fatigue were common symptoms in both COVID-19 and active AOSD patients. The distinct characteristics of AOSD included a higher proportion of skin rash (78.3% *vs*. 9.1%, P<0.001), arthralgia (73.9% *vs*. 21.8%, P<0.005), sore throat (65.2% *vs*. 14.5%, P<0.005), and liver dysfunction (43.5% *vs*. 10.9%, P<0.005). In contrast, COVID-19 patients had a higher proportion of pulmonary involvement (56.4% *vs*. 0.0%, p<0.001) and gastrointestinal symptoms (18.2% *vs*. 0.0%, p<0.05) compared with active AOSD patients. There were no significant differences in the age or female proportion between AOSD patients (mean age ± SD, 42.6 ± 13.5 years and 82.6%, respectively) and healthy subjects (40.2 ± 7.2 years and 80.6%, respectively).

**Table 1:** Demographic data and clinical manifestations in COVID-19 patients, active AOSD patients, and healthy subjects.

	COVID-19 (n = 55)	Active AOSD (n = 23)	Healthy control (n = 31)
Age, years	46.8 ± 16.0	42.6 ± 13.5	40.2 ± 7.2
Male proportion (%)	29 (52.7%)*	4 (17.4%)	6 (19.4%)
Female proportion (%)	26 (47.3%)	19 (82.6%)	25 (80.6%)
Fever	45 (81.8%)	21 (91.3%)	NA
Fatigue	23 (41.8%)	12 (52.2%)	NA
Myalgia	22 (40.0%)	13 (56.5%)	NA
Arthralgia	12 (21.8%)**	17 (73.9%)	NA
Skin rash	5 (9.1%)***	18 (78.3%)	NA
Sore throat	8 (14.5%)**	15 (65.2%)	NA
Liver dysfunction^a^	6 (10.9%)**	10 (43.5%)	NA
Lung involvement^b^	31 (56.4%)***	0 (0.0%)	NA
Gastrointestinal symptoms^c^	10 (18.2%)*	0 (0.0%)	NA
Headache	12 (21.8%)	2 (8.7%)	NA
Dysosmia	4 (7.3%)	0 (0.0%)	NA

Data are presented as mean ± SD or number (%); NA, not applicable; COVID-19, coronavirus disease 2019; AOSD, adult-onset Still’s disease.

^a^the presence of a twofold or more increase in alanine transaminase (ALT) that exceeded the upper limit of normal value (40 U/L).

^b^the presence of pneumonitis or interstitial lung lesions.

^c^the presence of nausea, vomiting, or diarrhea.

^*^p < 0.05, ^**^p < 0.005, ^***^p < 0.001, vs. active AOSD, as determined by chi-squared test.

### Plasma Levels of Gal-3, Gal-9, and sTIM-3 in COVID-19 Patients and AOSD Patients

**Figure 1:**
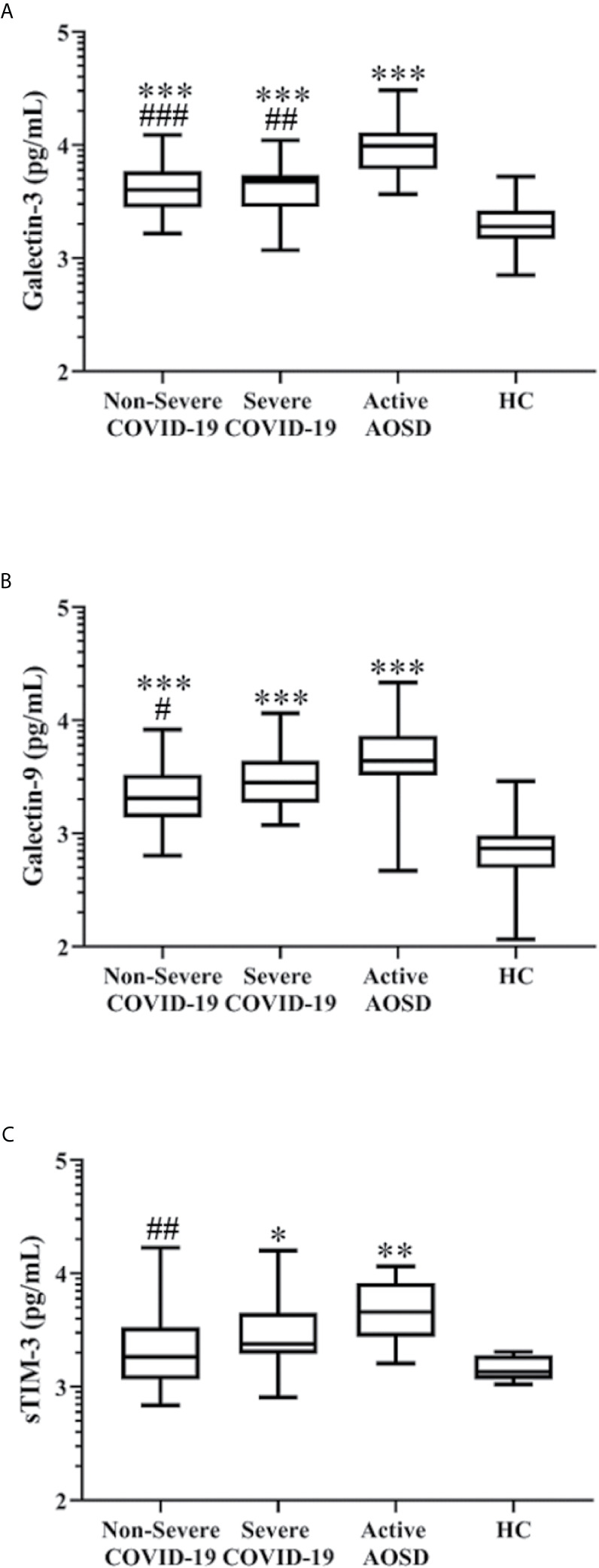
Comparison of plasma levels of Gal-3, Gal-9, and sTIM-3 in COVID-19, AOSD and HC. The difference in levels of Gal-3 **(A)**, Gal-9 **(B)**, and sTIM-3 **(C)** among non-severe COVID-19, severe COVID-19, active AOSD, and healthy control (HC). COVID-19, coronavirus disease 2019; AOSD, adult-onset Still’s disease; Gal-3, galectin-3; Gal-9, galectin-9; sTIM-3, soluble cell immunoglobulin and mucin-containing-molecule-3. Data are presented as logarithmic scale and box-plot diagrams, with the box encompassing the 25^th^ percentile (lower bar) to the 75^th^ percentile (upper bar). The horizontal line within the box indicates median value respectively for each group. *p-value < 0.05, **p-value < 0.01, ***p-value < 0.001, *versus* HC; ^#^p-value < 0.05, ^##^p-value < 0.01, ^###^p-value < 0.001, *versus* AOSD patients, determined by the Kruskal-Wallis test with a *post-hoc* Dunn’s test.

As shown in **Figure 1A** and **Table 2**, Gal-3 levels were significantly higher in non-severe COVID-19, severe COVID-19, and active AOSD patients compared with HC (all p<0001). Active AOSD patients also have significantly higher Gal-3 levels than non-severe or severe COVID-19 patients, while there was no significant difference in Gal-3 levels between non-severe and severe COVID-19 patients. Similarly, Gal-9 levels were significantly higher in non-severe COVID-19, severe COVID-19, and active AOSD patients compared with HC. Gal-9 levels were also significantly higher in active AOSD patients than those in non-severe COVID-19 patients, while no significant difference in Gal-9 levels between active AOSD and severe COVID-19 or between non-severe and severe COVID-19 patients (**Figure 1B**). Plasmas sTIM-3 levels were significantly higher in severe COVID-19 patients and active AOSD patients compared to HC. AOSD patients also have significantly higher sTIM-3 levels than non-severe COVID-19 patients, but no significant difference in sTIM-3 levels between active AOSD and severe COVID-19 or between non-severe and severe COVID-19 patients (**Figure 1C**).

**Table 2:** Plasma levels of galectins, cytokine profiles, and ferritin in COVID-19 patients, active AOSD patients, and healthy subjects.

	Non-severe COVID-19 (n = 31)	Severe COVID-19 (n = 24)	Active AOSD (n = 23)	Healthy subjects (n = 31)
Gal-3 levels, pg/mL	4002 (2790-5866)^***,###^	4723 (2818-5424)^***,##^	9820 (6200-12930)^***^	1897 (1477-2629)
Gal-9 levels, pg/mL	2044 (1385-3303)^***,#^	2811 (1866-4371)^***^	4370 (3249-7311)^***^	739 (495-960)
sTIM-3 levels, pg/mL	1838 (1161-3360)^##^	2393 (1954-4499)^*^	4563 (2759-8240)^**^	1351 (1164-1897)
IL-1β levels, pg/mL	6.1 (1.8-12.9)^*^	8.8 (4.5-27.0)^***^	6.13 (3.6-12.8)^**^	1.7 (1.0-12.6)
IL-1Ra levels, pg/mL	9.0 (4.77-15.6)^***^	13.5 (8.2-41.4)^***^	15.6 (8.8-25.2)^***^	0.8 (0.01-2.5)
IL-10 levels, pg/mL	0.6 (0.3-8.42)^#,$^	6.5 (1.0-15.3)^***^	19.0 (1.6-88.8)^***^	0.6 (0.2-0.6)
IFN-α2 levels, pg/mL	5.9 (12.7-20.26)^#,$ $^	35.7 (14.5-105)^***^	29.5 (18.4-69.4)^***^	2.7 (2.7-10.8)
IFN-γ levels, pg/mL	1.5 (0.7-3.87)^###^	3.2 (0.8-10.5)	17.9 (5.0-45.8)^**^	1.8 (1.0-6.1)
IL-17A levels, pg/mL	1.7 (0.7-4.2)^###^	3.7 (1.83-11.02)	14.3 (6.5-307.7)^**^	2.0 (1.7-3.8)
IL-6 levels, pg/mL	2.4 (0.6-4.5)^***,#^	13.8 (3.68-29.98)^***^	35.6 (2.2-1500)^***^	0.1 (0.02-0.3)
TNF-α levels, pg/mL	18.3 (11.0-29.3)^***,##^	29.1 (19.6-70.2)^***^	54.0 (27.3-27.3)^***^	7.2 (4.5-12.2)
IL-18 levels, pg/mL	29.9 (18.2-46.0)^**,###^	40.4 (31.1-69.4)^***,###^	2768 (966.1-6754)^***^	6.1 (4.3-12.3)
Ferritin levels, ng/mL	209 (191-240)^***,###^	220 (195-240)^***,###^	1111 (382.4-4200)^***^	57 (53-59)

Data are presented as median (25^th^ -75^th^ quartile range); COVID-19, coronavirus disease 2019; AOSD, adult-onset Still’s disease; Gal-3, galectin-3; Gal-9, galectin-9; sTIM-3, soluble cell immunoglobulin and mucin-containing-molecule-3; IFN, interferon; IL, interleukin; IL-1Ra, interleukin-1 receptor antagonist; TNF-α, tumor necrosis factor-α.

^*^p < 0.05, ^**^p < 0.01, ^***^p < 0.001, vs. healthy subjects, as determined by Kruskal-Wallis test using a post-hoc Dunn’s test.

^#^p < 0.05, ^##^p < 0.01, ^###^p < 0.001, vs. active AOSD, as determined by Kruskal-Wallis test using a post-hoc Dunn’s test.

^$^p < 0.05, ^$$^p < 0.01, non-severe COVID-19 vs. severe COVID-19, as determined by Kruskal-Wallis test using a post-hoc Dunn’s test.

Among COVID-19 patients, significantly higher Gal-3, Gal-9, and sTIM-3 were observed in purchased plasma samples compared with plasma samples from Chinese patients (**Supplementary Table 1**).

### Plasma Levels of Cytokine Profiles and Ferritin in COVID-19 and AOSD Patients

**Figure 2:**
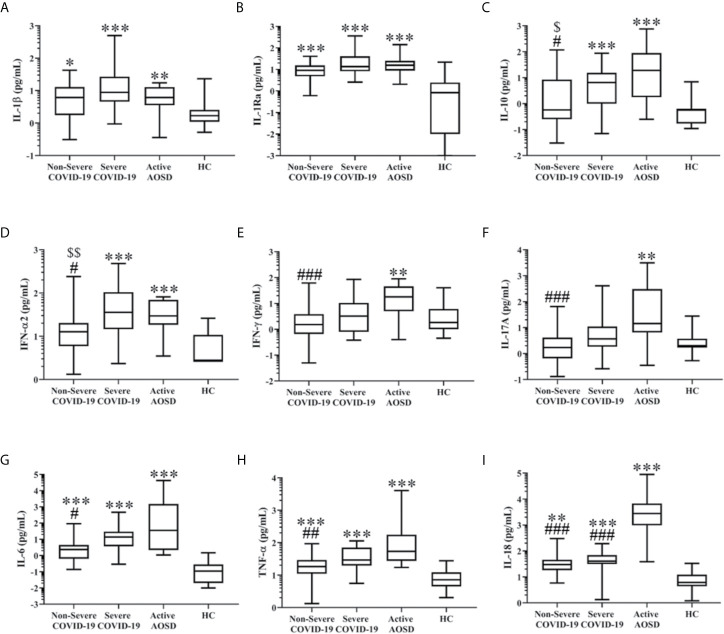
Comparison of plasma levels of cytokine profiles in COVID-19, AOSD and HC. The difference in levels of IL-1β **(A)**, IL-1Ra **(B)**, IL-10 **(C)**, IFN-α2 **(D)**, IFN-γ **(E)**, IL-17A **(F)**, IL-6 **(G)**, TNF-α **(H)**, and IL-18 **(I)** among non-severe COVID-19, severe COVID-19, active AOSD, and healthy control (HC). COVID-19, coronavirus disease 2019; AOSD, adult-onset Still’s disease; IFN, interferon; IL, interleukin; IL-1Ra, interleukin-1 receptor antagonist; TNF-α, tumor necrosis factor-α. Data are presented as a logarithmic scale and box-plot diagrams, with the box encompassing the 25^th^ percentile (lower bar) to the 75^th^ percentile (upper bar). The horizontal line within the box indicates median value respectively for each group. *p-value < 0.05, **p-value < 0.01, ***p-value < 0.001, *versus* HC; ^#^p-value < 0.05, ^##^p-value < 0.01, ^###^p-value < 0.001, *versus* AOSD patients; ^$^p-value < 0.05, ^$$^p< 0.01, *versus* severe COVID-19 patients, determined by the Kruskal-Wallis test with a *post-hoc* Dunn’s test.

As shown in **Table 2** and **Figures 2A, B**, IL-1β and IL-1Ra levels were significantly higher in COVID-19 patients and active AOSD patients compared with HC, but there was no significance in IL-1β or IL-1Ra levels between COVID-19 and active AOSD patients. In **Figures 2C, D**, severe COVID-19 and active AOSD patients had significantly higher IL-10 and IFN-α2 compared with HC or non-severe COVID-19 patients. As shown in **Figures 2E, F**, IFN-γ and IL-17A levels were significantly higher in active AOSD patients compared with HC (both p<0.01) or non-severe COVID-19 patients (both p<0.001), but there was no significant difference in IFN-γ or IL-17A levels between COVID-19 patients and HC. Plasma IL-6 and TNF-α levels were significantly higher in COVID-19 patients and active AOSD patients than in HC (**Figures 2G, H**). Active AOSD patients also had significantly higher levels of IL-6 and TNF-α than non-severe COVID-19 patients. In **Figure 2I**, active AOSD patients and COVID-19 patients had significantly higher IL-18 levels than HC, with the levels even higher in active AOSD compared with severe COVID-19 patients. As shown in **Table 2**, ferritin levels were significantly higher in COVID-19 patients and active AOSD patients than in HC (all p<0.001). Active AOSD had significantly higher ferritin levels compared with COVID-19, but there was no significant difference in ferritin levels between non-severe and severe COVID-19. As illustrated in **Supplementary Table 1**, significantly higher IFN-α2, IL-10, and IL-6 levels were observed in purchased plasma samples compared with samples from Chinese patients.

### Association of Galectins and Cytokine Profiles With Clinical Features in AOSD

A logistic regression analysis was used to evaluate the simultaneous effects of galectins and cytokine profiles on the occurrence of clinical features in AOSD patients. As illustrated in **Supplementary Table 2**, IL-18 was a significant predictor of myalgia (p<0.05) and a probable predictor of liver dysfunction (p=0.096).

### Distinct Markers That Differentiate Active AOSD From Severe COVID-19

**Figure 3:**
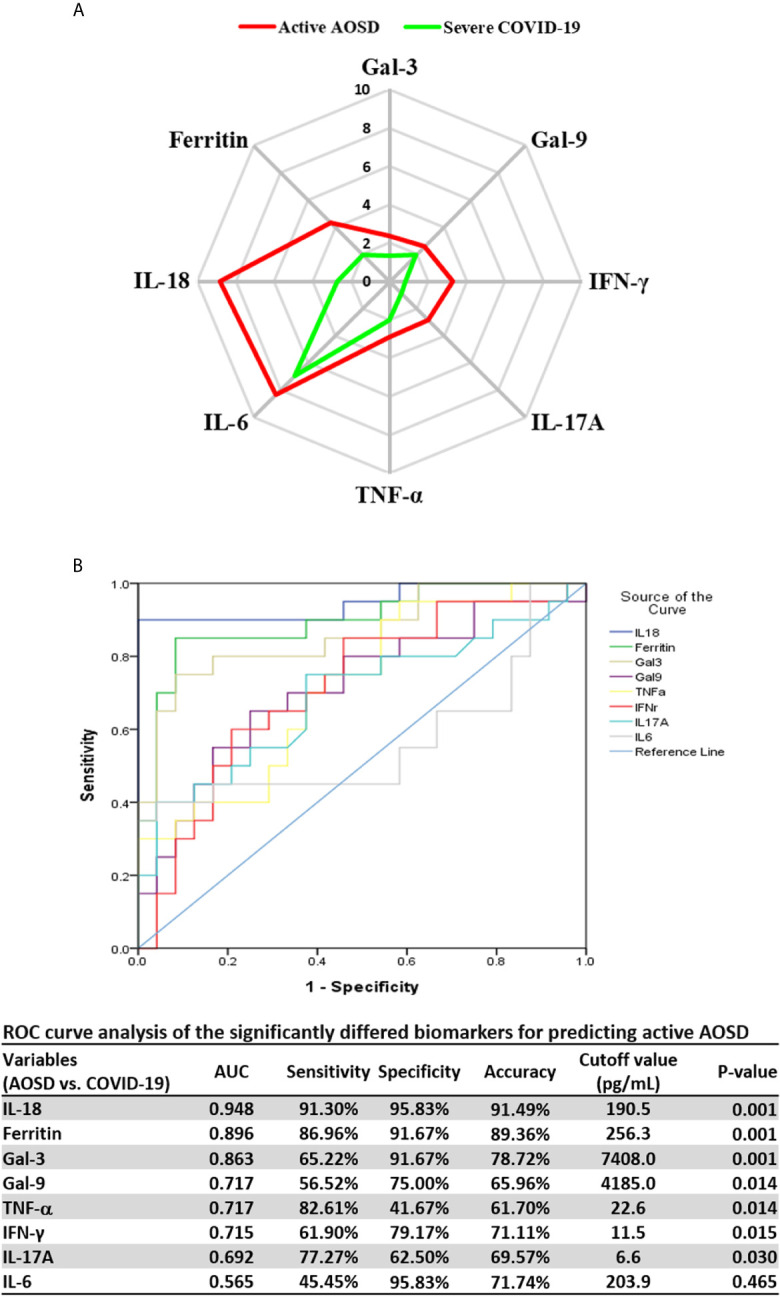
Significantly different galectins and cytokines between active AOSD and severe COVID-19, and ROC curves analysis. **(A)** The significantly different levels of galectins and cytokine profiles shown by radar charts between active AOSD and severe COVID-19. **(B)** The ROC curves analysis of the significantly differed biomarkers for predicting active AOSD. The illustrated levels in **(A)** were presented as Log2 fold changes of markers, defined as the median expression level ratio of active AOSD or severe COVID-19 to healthy subjects. AOSD, adult-onset Still’s disease; COVID-19, coronavirus disease 2019; ROC, receiver-operating characteristic; AUC, area under ROC curve.

To illustrate the significant biomarkers which differentiate active AOSD from severe COVID-19, we used a radar chart to depict galectins, cytokine profiles, and ferritin levels (**Figure 3A**). The levels were presented as the Log2 fold changes of markers, defined as the median expression level ratio of active AOSD or severe COVID-19 to healthy controls (HC). Compared with HC, IL-18, IL-6, and ferritin were markedly elevated in active AOSD patients (Log2 fold changes, 8.86, 8.34, and 4.30, respectively) and in severe COVID-19 patients (Log2 fold changes, 2.73, 6.97, and 1.97, respectively). Compared with severe COVID-19 patients, active AOSD patients had 68-fold higher levels of IL-18 and 5-fold higher levels of ferritin (both p<0.001).

The ROC analysis of the putative biomarkers revealed that IL-18 levels at the cut-off value 190.5pg/mL had the highest discriminative power with AUC of 0.948, the sensitivity of 91.3%, specificity of 95.8%, and an accuracy of 91.5% for differentiating active AOSD from severe COVID-19 (**Figure 3B**).

### Logistic Regression Analysis for Predicting Active AOSD

Given our primary goal to compare the differences in the components of cytokine storm between active AOSD and severe COVID-19, a logistic regression analysis was used to identify the cytokine biomarkers for predicting AOSD. As illustrated in **Table 3**, the univariate regression analysis identified female gender, IFN-γ, IL-6, IL-17A, IL-18, and TNF-α as the potential predictors of active AOSD, and multivariate analysis demonstrated IL-18 as a significant predictor for active AOSD.******

**Table 3:** Logistic regression analysis of cytokine profiles to predict active AOSD in the studied cohort.

Baseline variables	Univariate model				Multivariate model			
	OR	95%CI		*p* value	OR	95%CI		*p* value
Age	0.98	0.95-1.01)		0.262				
Gender								
Male	ref.							
Female	5.30	(1.59-17.61)		0.007				
IFN-α2 level	1.00	(0.99-1.01)		0.765				
IFN-γ level	1.04	(1.01-1.07)		0.003				
IL-1β level	0.97	(0.92-1.02)		0.231				
IL1RA level	1.00	(0.99-1.01)		0.845				
IL-6 level	1.00	(1.00-1.01)		0.036				
IL-10 level	1.01	(1.00-1.03)		0.100				
IL-17A level	1.01	(1.00-1.01)		0.016				
IL-18 level	1.01	(1.00-1.02)		0.011	1.01	(1.00-1.01)		0.030
TNF-α level	1.02	(1.01-1.04)		0.007				

OR, Odds ratio; 95% CI, 95% confidence interval; IFN, interferon; IL, interleukin; TNF-α, tumor necrosis factor-α.

## Discussion

With hyperinflammation and some clinical manifestations common to both severe COVID-19 and active AOSD, it is an unmet need to identify biomarkers that can differentiate between these two diseases. Although most of the cytokines examined herein were elevated in both diseases compared with healthy subjects (HC), active AOSD patients had significantly higher levels of IFN-γ, IL-17A, IL-18, and ferritin than COVID-19 patients. Gal-3 and Gal-9 have recently been found to play crucial roles in the pathogenesis of COVID-19 (27, 28) and AOSD (30, 33), and our study is the first to reveal higher Gal-3, Gal-9, and sTIM-3 levels in active AOSD compared with COVID-19 patients. Despite the similarities in clinical and laboratory features in COVID-19 and AOSD, we are the first to identify IL-18 as a potential discriminator between active AOSD and severe COVID-19, with a high AUC (0.948), high sensitivity, and high specificity, as well as a significant predictor of active AOSD. However, our results should be confirmed by future large-scale prospective studies.

In this study, the demographic data of COVID-19 patients were similar to those in other previous studies, showing that older age and male gender were the risk factors for the occurrence or severity of COVID-19 (1, 2, 35, 41). In comparison, the clinical manifestations of skin rash, arthralgia or arthritis, sore throat, and liver dysfunction were more common in our AOSD patients. In contrast, the respiratory and gastrointestinal symptoms, which were rarely seen in AOSD, were prominent features of COVID-19 patients.

Consistent with the previous reports (27, 28), we revealed significantly higher Gal-3 levels in COVID-19 and active AOSD patients compared with HC. Given that the immune cells can release Gal-3 in inflammatory responses (42), we speculate that Gal-3 can serve as a potential biomarker of hyperinflammation in COVID-19 and AOSD. Similarly, elevated Gal-9 levels were observed in both COVID-19 patients and active AOSD patients, as also found in previous studies (28, 33). Seki et al. revealed that Gal-9 negatively regulates proinflammatory T-cell responses (31) by inducing apoptosis of Th1 or Th17 cells, which play an important role in COVID-19 (28) and AOSD (17).

In response to SARS-COV-2 infection, an exaggerated immune response with inflammatory cytokines overproduction developed in COVID-19 (3–6, 8, 28). Our COVID-19 patients had significantly higher levels of IL-1β, IL-1Ra, IL-10, IFN-α2, IL-6, IL-18, and TNF-α than HC. Since SARS-CoV-2-triggered inflammation can activate NLRP3-inflammasome (6) and elevated NLRP3-inflammasome levels are a feature of AOSD (19), increased IL-1β levels were observed in both diseases. Similar to a recent report (35), there was no significant difference in IL-1β levels between COVID-19 and active AOSD patients in this study. IL-1Ra, an inhibitory cytokine that controls inflammatory responses, plays a critical role in cytokine storm in active AOSD or COVID-19. An attenuated form of IL-1Ra, anakinra, is currently used to treat AOSD or COVID-19 (20, 43). Like IL-1Ra, IL-10 likely exerts an inhibitory effect on hyperinflammation, evidenced by elevated IL-10 levels in active AOSD and severe COVID-19. Our severe COVID-19 patients also had significantly higher levels of IL-10 and IL-1Ra compared with non-severe patients, which was also shown in a previous report (44). The compensatory roles of both inhibitory cytokines might reflect a shared phenomenon in the pathogenesis of inflammatory diseases characterized by cytokine storms like COVID-19 and active AOSD.

The levels of IFN-γ, a Th1-derived cytokine that contributes to inflammation amplification, were increased in our active AOSD and were higher than those in severe COVID-19. Given a protective role of IFN-γ against viral infection, low IFN-γ levels may cause an excessive viral replication and trigger hyperinflammation in severe COVID-19. As in the previous report (17), elevated IL-17A levels were observed in AOSD patients, even higher than those in severe COVID-19. Although an increased capacity of T cells to produce IL-17A may occur in COVID-19 pneumonia (28), there was no significant elevation of IL-17A in our COVID-19 patients. This discrepancy may be related to the difference in the enrolled COVID-19 patients’ characteristics and blood sampling timing among the different studies.

In the present study, IL-6 levels were significantly higher in severe COVID-19 and active AOSD patients compared with HC, suggesting uncontrolled amplification of cytokine production. Severe COVID-19 patients had significantly higher IL-6 levels than non-severe patients, suggesting a pathogenic role of IL-6 in a cytokine storm. Along the same lines, therapeutics targeting IL-6 signaling, including the IL-6 receptor antagonist tocilizumab (TCZ), showed promising results in treating severe COVID-19 (45). Meanwhile, active AOSD patients had even higher IL-6 levels than severe COVID-19 patients. A recent meta-analysis suggested that TCZ is an effective biological agent for AOSD treatment (46). In contrast, TCZ therapy resulted in limited clinical improvement in COVID-19 patients at day28, according to a meta-analysis of randomized controlled trials (47).

SARS-CoV-2-triggered inflammation may activate NLRP3-inflammasome with overproduction of IL-18 (6), a phenomenon observed in our COVID-19 patients. Active AOSD patients similarly had elevated expression of NLRP3-inflammasome signaling (19). Interestingly, our active AOSD patients had 68-fold higher levels of IL-18 than severe COVID-19 patients. Among the cytokines involved, IL-18 was a significant predictor of active AOSD and its myalgia. Besides, IL-18 showed the highest discriminating ability between AOSD and COVID-19 in the ROC analysis of the putative markers. These findings suggest that exaggerated production of IL-18 is highly characteristic of active AOSD, resonating with previous reports showing IL-18 as a diagnostic marker and indicator of disease activity in AOSD (16, 48, 49). Blocking IL-18 with recombinant IL18 BP (tadekinig alfa) has therapeutic efficacy for AOSD (22) but has yet to be applied to COVID-19 treatment now.

Beyond its iron storage role, ferritin participates in the pathogenesis of inflammation (50) and may stimulate inflammatory pathways to amplify the inflammatory process (51). In response to viral infection, ferritin synthesis can be upregulated by the inflammatory cytokines (52). In our study, both severe COVID-19 and active AOSD patients showed elevated ferritin levels, supporting the proposition that they belong to the group of “hyperferritinemic syndrome” (23). Interestingly, 5-fold higher ferritin levels were observed in our active AOSD patients than in severe COVID-19, which is consistent with the analysis results reported by Meng et al. (35) and Colafrancesco et al. (23). Therefore, ferritin levels may have a great ability to help discriminate AOSD from COVID-19.

Despite the novel findings, there are some limitations of our study. The lack of a significant difference in ferritin and IFN-γ levels between severe and non-severe COVID-19 patients might be due to the small sample size. Because this is a cross-sectional study, we do not have serial data of galectins or cytokine profiles over time. Besides, the timing of blood collection from COVID-19 patients may not be during the acute infection phase. Therefore, future studies enrolling more COVID-19 and AOSD patients and investigating the biological role of galectins signaling pathway in the cytokine storms’ pathogenesis are certainly needed.

In conclusion, both active AOSD and severe COVID-19 patients showed elevated Gal-3, Gal-9, and cytokines, including IL-1β, IL-1Ra, IL-10, IL-6, IL-18, and TNF-a, supporting a common link of cytokine storm in the pathogenesis of both diseases. Compared with severe COVID-19 patients, active AOSD patients had markedly higher levels of IL-18, which is a potential discriminator between active AOSD and severe COVID-19. The distinct cytokine profiles might be linked to different clinical manifestations and therapeutic responses to cytokine-targeted agents in both diseases. However, a clear distinction between severe COVID-19 and active AOSD is challenging and needs to be explored in future studies.

## Data Availability Statement

The original contributions presented in the study are included in the article/**Supplementary Material**. Further inquiries can be directed to the corresponding author.

## Ethics Statement

The studies involving human participants were reviewed and approved by Research Ethics Committee, China Medical University & Hospital, Taichung, Taiwan. The patients/participants provided their written informed consent to participate in this study.

## Author Contributions 

P-KC conceived and designed the study, acquired the laboratory data, performed the data analysis, and drafted the manuscript. J-LL and P-HH acquired the clinical data and performed the data analysis. J-LH conducted the experiments and performed data analysis. C-KC, NT, and H-JL conducted the experiments. D-YC conceived and designed the study, acquired the clinical data, performed data analysis, and revised the manuscript. All authors contributed to the article and approved the submitted version.

## Funding

This work was supported by a grant from China Medical University Hospital (DMR-110-021), China Medical University (1095310A), and by a grant (MOST 107-2314-B-039-053-MY3) from the Ministry of Science and Technology, Taiwan.

## Conflict of Interest

The authors declare that the research was conducted in the absence of any commercial or financial relationships that could be construed as a potential conflict of interest.

## Publisher’s Note

All claims expressed in this article are solely those of the authors and do not necessarily represent those of their affiliated organizations, or those of the publisher, the editors and the reviewers. Any product that may be evaluated in this article, or claim that may be made by its manufacturer, is not guaranteed or endorsed by the publisher.

## Abbreviations

AOSD, adult-onset Still’s disease; AUC, Area under the receiver operating characteristic curve; COVID-19, Coronavirus Disease 2019; CRP, C-reactive protein; DAMP, damage associated with the molecular pattern; HC, healthy controls; Gal, Galectins; IL, interleukin; IL-1Ra, interleukin-1 receptor antagonist; IFN, Interferon; MAS, macrophage activation syndrome; NLRP3, NOD, LRR, and pyrin domain-containing protein 3; TNF-α, tumor necrosis factor-α; ROC, Receiver operating characteristic; SARS-CoV-2, severe acute respiratory syndrome coronavirus 2; sTIM-3, soluble cell immunoglobulin and mucin-containing-molecule-3; Th1, type 1 helper T cell; TCZ, tocilizumab.
