# Supplementary material for: Interleukin-18 Is a Potential Biomarker to Discriminate Active Adult-Onset Still’s Disease From COVID-19
Source: Front Immunol. 2021 Jul 23;12:719544. doi: 10.3389/fimmu.2021.719544 (PMC8343229; doi:10.3389/fimmu.2021.719544)
Supplement: Supplementary file 1 [file Table_1.docx]

**Supplementary Table1**. Comparison of galectins, cytokine profiles and ferritin levels in the purchased plasma samples from COVID-19 patients and samples from Chinese COVID-19 patients.

|  | Purchased plasma samples from patients with  COVID-19 (n=25) | | Plasma samples from  Chinese patients with  COVID-19 (n=30) | |
| --- | --- | --- | --- | --- |
| The proportion of  severe COVID-19 (%) | | 15 (60.0%)^$^ | | 9 (30.0%) |
| Gal-3 levels, pg/mL | 5257 (2948-7899)^*^ | | 3814 (2789-4828) | |
| Gal-9 levels, pg/mL | 3354 (1910-5069)^**^ | | 2061 (1353-2723) | |
| sTIM-3 levels, pg/mL | 3144 (1969-5814)^***^ | | 1517 (1084-2312) | |
| IFN-α2 levels, pg/mL | 24.1 (13.6-45.2)^*^ | | 11.7 (5.8-18.2) | |
| IFN-γ levels, pg/mL | 1.7 (0.6-5.4) | | 2.5 (1.1-7.2) | |
| IL-17A levels, pg/mL | 3.0 (1.6-7.9) | | 1.8 (0.6 -5.4) | |
| IL-10 levels, pg/mL | 7.4 (1.0-19.8)^***^ | | 0.5 (0.19-3.02) | |
| IL-1Ra levels, pg/mL | 10.8 (5.8 -28.8) | | 10.5 (5.0-17.6) | |
| IL-1β levels, pg/mL | 6.8 (4.7-11.9) | | 10.0 (1.6-23.3) | |
| IL-6 levels, pg/mL | 10.5 (3.6-25.7)^**^ | | 1.6 (0.6 -6.9) | |
| TNF-α levels, pg/mL | 20.1 (15.2-37.9) | | 24.5 (16.1-42.5) | |
| IL-18 levels, pg/mL | 40.6 (26.6-69.3) | | 30.7 (18.3-46.7) | |
| Ferritin levels, ng/mL | 223 (204-250)^*^ | | 203 (188-235) | |

Data are presented as number (%) or median (25^th^ -75^th^ quartile range); COVID-19: coronavirus disease 2019; Gal-3: galectin-3; Gal-9: galectin-9; sTIM-3: soluble cell immunoglobulin and mucin-containing-molecule-3; IFN: interferon; IL: interleukin; IL-1Ra: interleukin-1 receptor antagonist; TNF-α: tumor necrosis factor-α.

^$^p<0.05, vs. Chinese patients with COVID-19, as determined by chi-squared test.

^*^p<0.05, ^**^p<0.01, ^***^p<0.001, vs. Chinese patients with COVID-19, as determined by Mann-Whitney test.
