# Supplementary material for: Interleukin-18 Is a Potential Biomarker to Discriminate Active Adult-Onset Still’s Disease From COVID-19
Source: Front Immunol. 2021 Jul 23;12:719544. doi: 10.3389/fimmu.2021.719544 (PMC8343229; doi:10.3389/fimmu.2021.719544)
Supplement: Supplementary file 2 [file Table_2.docx]

**Supplementary Table 2. Associations of galectins and cytokine profiles with clinical features in active AOSD patients**

| Variable | **Fatigue** | | |  | **Myalgia** | | |  | **Arthralgia** | | |  | **Skin rash** | | |  | **Sore Throat** | | |  | **Liver Dysfunction** | | |
| --- | --- | --- | --- | --- | --- | --- | --- | --- | --- | --- | --- | --- | --- | --- | --- | --- | --- | --- | --- | --- | --- | --- | --- |
|  | OR | 95% CI | p-value |  | OR | 95% CI | p-value |  | OR | 95% CI | p-value |  | OR | 95% CI | p-value |  | OR | 95% CI | p-value |  | OR | 95% CI | p-value |
| Gal-3 | 1.000 | 0.9998 - 1.000 | 0.353 |  | 1.000 | 1.000 - 1.000 | 0.085 |  | 1.000 | 0.9999 - 1.000 | 0.317 |  | 1.000 | 0.9999 - 1.000 | 0.413 |  | 1.000 | 0.9999 - 1.000 | 0.4917 |  | 1.000 | 1.000 - 1.000 | 0.172 |
| Gal-9 | 1.000 | 0.9998 - 1.000 | 0.898 |  | 1.000 | 1.000 - 1.001 | 0.133 |  | 1.000 | 1.000 - 1.001 | 0.183 |  | 1.000 | 1.000 - 1.001 | 0.190 |  | 1.000 | 0.9998 - 1.000 | 0.8649 |  | 1.000 | 0.9999 - 1.000 | 0.282 |
| sTIM-3 | 1.000 | 0.9998 - 1.001 | 0.529 |  | 1.000 | 0.9998 - 1.001 | 0.443 |  | 1.000 | 0.9995 - 1.000 | 0.687 |  | 1.002 | 1.000 - 1.010 | 0.206 |  | 1.000 | 0.9998 - 1.001 | 0.5443 |  | 1.000 | 0.9996 - 1.000 | 0.933 |
| IFN-α2 | 1.002 | 0.9525 - 1.052 | 0.926 |  | 1.026 | 0.9800 - 1.084 | 0.291 |  | 1.067 | 1.003 - 1.218 | 0.124 |  | 1.008 | 0.9612 - 1.066 | 0.741 |  | 1.064 | 1.002 - 1.204 | 0.1248 |  |  |  |  |
| IFN-γ | 1.009 | 0.9740 - 1.050 | 0.608 |  | 1.017 | 0.9809 - 1.063 | 0.381 |  | 1.015 | 0.9756 - 1.069 | 0.491 |  | 1.054 | 0.9964 - 1.166 | 0.152 |  | 1.029 | 0.9888 - 1.083 | 0.2079 |  | 1.013 | 0.9772 - 1.053 | 0.483 |
| IL-17A | 1.003 | 1.000 - 1.010 | 0.224 |  | 1.001 | 0.9993 - 1.003 | 0.444 |  | 1.000 | 0.9989 - 1.003 | 0.803 |  | 1.000 | 0.9987 - 1.002 | 0.963 |  | 1.002 | 0.9996 - 1.008 | 0.356 |  | 1.001 | 0.9994 - 1.003 | 0.343 |
| IL-10 | 1.004 | 0.9980 - 1.014 | 0.273 |  | 0.999 | 0.9910 - 1.005 | 0.748 |  | 0.998 | 0.9890 - 1.003 | 0.423 |  | 1.037 | 0.9992 - 1.173 | 0.395 |  | 0.998 | 0.9891 - 1.003 | 0.4274 |  | 1.000 | 0.9923 - 1.006 | 0.881 |
| IL-1Ra | 0.979 | 0.8767 - 1.012 | 0.428 |  | 1.006 | 0.9811 - 1.036 | 0.603 |  | 1.001 | 0.9756 - 1.031 | 0.951 |  | 0.995 | 0.9684 - 1.023 | 0.665 |  | 0.999 | 0.9737 - 1.028 | 0.9524 |  | 1.043 | 1.007 - 1.213 | 0.149 |
| IL-1β | 0.950 | 0.8073 - 1.106 | 0.510 |  | 1.119 | 0.9549 - 1.350 | 0.189 |  | 1.129 | 0.9394 - 1.438 | 0.239 |  | 1.011 | 0.8412 - 1.238 | 0.906 |  | 1.097 | 0.9314 - 1.334 | 0.2931 |  | 0.974 | 0.8288 - 1.137 | 0.741 |
| IL-6 | 1.001 | 1.000 - 1.003 | 0.152 |  | 1.000 | 0.9999 - 1.000 | 0.715 |  | 1.000 | 0.9998 - 1.000 | 0.350 |  | 1.000 | 0.9998 - 1.000 | 0.263 |  | 1.000 | 1.000 - 1.001 | 0.4642 |  | 1.000 | 0.9999 - 1.000 | 0.951 |
| TNF-α | 1.011 | 1.001 - 1.033 | 0.217 |  | 1.000 | 0.9978 - 1.001 | 0.581 |  | 0.999 | 0.9968 - 1.000 | 0.305 |  | 0.999 | 0.9965 - 1.000 | 0.261 |  | 1.001 | 0.9994 - 1.005 | 0.5363 |  | 1.000 | 0.9981 - 1.001 | 0.836 |
| IL-18 | 1.000 | 1.000 - 1.000 | 0.348 |  | 1.001 | 1.000 - 1.002 | 0.045* |  | 1.000 | 1.000 - 1.001 | 0.175 |  | 1.000 | 1.000 - 1.001 | 0.201 |  | 1.000 | 0.9999 - 1.000 | 0.6413 |  | 1.002 | 1.001 - 1.006 | 0.096 |

AOSD: adult-onset Still’s disease; Gal-3: galectin-3; Gal-9: galectin-9; sTIM-3: soluble cell immunoglobulin and mucin-containing-molecule-3; IFN: interferon; IL: interleukin; IL-1Ra: interleukin-1 receptor antagonist; TNF-α: tumor necrosis factor-α. Liver dysfunction was defined as the presence of a twofold or more increase in alanine transaminase (ALT) that exceeded the upper limit of normal value (40 U/L). p-values were determined by the logistic regression analysis.
